# Serum Metabolomic Profiles of Paratuberculosis Infected and Infectious Dairy Cattle by Ambient Mass Spectrometry

**DOI:** 10.3389/fvets.2020.625067

**Published:** 2021-01-20

**Authors:** Alessandra Tata, Ivana Pallante, Andrea Massaro, Brunella Miano, Massimo Bottazzari, Paola Fiorini, Mauro Dal Prà, Laura Paganini, Annalisa Stefani, Jeroen De Buck, Roberto Piro, Nicola Pozzato

**Affiliations:** ^1^Istituto Zooprofilattico delle Venezie (IZSVe), Legnaro, Italy; ^2^Department of Production Animal Health, University of Calgary, Calgary, AB, Canada

**Keywords:** metabolomic, paratuberculosis, biomarker, cattle, DART-MS, Jonhe's disease, *mycobacterium avium* subsp *paratubercolosis*

## Abstract

*Mycobacterium avium* subsp. paratuberculosis (MAP) is the causative agent of paratuberculosis [Johne's disease (JD)], a chronic disease that causes substantial economic losses in the dairy cattle industry. The long incubation period means clinical signs are visible in animals only after years, and some cases remain undetected because of the subclinical manifestation of the disease. Considering the complexity of JD pathogenesis, animals can be classified as infected, infectious, or affected. The major limitation of currently available diagnostic tests is their failure in detecting infected non-infectious animals. The present study aimed to identify metabolic markers associated with infected and infectious stages of JD. Direct analysis in real time coupled with high resolution mass spectrometry (DART-HRMS) was, hence, applied in a prospective study where cohorts of heifers and cows were followed up annually for 2–4 years. The animals' infectious status was assigned based on a positive result of both serum ELISA and fecal PCR, or culture. The same animals were retrospectively assigned to the status of infected at the previous sampling for which all JD tests were negative. Stored sera from 10 infected animals and 17 infectious animals were compared with sera from 20 negative animals from the same herds. Two extraction protocols and two (-/+) ionization modes were tested. The three most informative datasets out of the four were merged by a mid-level data fusion approach and submitted to partial least squares discriminant analysis (PLS-DA). Compared to the MAP negative subjects, metabolomic analysis revealed the m/z signals of isobutyrate, dimethylethanolamine, palmitic acid, and rhamnitol were more intense in infected animals. Both infected and infectious animals showed higher relative intensities of tryptamine and creatine/creatinine as well as lower relative abundances of urea, glutamic acid and/or pyroglutamic acid. These metabolic differences could indicate altered fat metabolism and reduced energy intake in both infected and infectious cattle. In conclusion, DART-HRMS coupled to a mid-level data fusion approach allowed the molecular features that identified preclinical stages of JD to be teased out.

## Introduction

Bovine paratuberculosis, known as Johne's disease (JD), is a chronic infectious disease of ruminants resulting in diarrhea, wasting, weight loss, emaciation, and eventual death (1–3). The etiologic agent of JD is *Mycobacterium avium* subsp. *paratuberculosis* (MAP), a slow growing, obligate intracellular pathogen (4).

JD is widespread worldwide, causes great economic losses, and is mainly transmitted through the fecal-oral route (2, 5, 6). The susceptibility is age-dependent, with young calves highly sensitive to infection. The susceptibility decreases in heifers, with adult cows being infected only in the case of high infectious doses or long exposure time (2, 5, 7). In JD, animals can be classified into three groups: infected when MAP is present intracellularly in the animal, infectious when the animal is shedding MAP via feces and affected when clinical signs are visible (8). Common diagnostic tests, such as fecal Polymerase Chain Reaction (PCR) and enzyme-linked immunosorbent assay (ELISA) lack of sensitivity in earlier stages of infection, characterized by intermittent MAP shedding and absence of specific Th-2 response (9). The biology of MAP infection, the long incubation period, the pathogenesis, and the difficulties in detecting infected animals in the absence of accurate diagnostic tests entail delayed diagnosis of JD, so disease control in infected herds is a challenge (10).

Over recent years, genomics, transcriptomics and proteomics were introduced to overcome the lack of efficient diagnostic methods in the preclinical stages of the disease (11–16); the aim was to identify specific pathways related to disease progression through the study of the expression of genes and proteins. In this way, biomarkers related to MAP infection can be determined (11, 14–16). In addition, metabolomics has emerged as a means to identify altered metabolic pathways in infected animals, by characterizing the metabolic profiles of cattle experimentally infected with MAP (13). Serum lipidomics of control and MAP infected cattle were also explored, utilizing high-resolution mass spectrometry, with the data showing that altered availability of choline-containing lipids occurs late in the disease process, and it is most likely a result of malnutrition and altered biosynthetic capacities of the liver and the gastrointestinal tract (17). Volatile organic signatures of breath were defined by gas-chromatography to differentiate healthy cattle from MAP infected cattle (18). In human medicine, the metabolomic approach was used to differentiate latent infection caused by *Mycobacterium tuberculosis* from active tuberculosis. The identification of mycobacterial biomarkers of infection could contribute to faster and more accurate diagnosis than the tuberculin skin or interferon gamma tests (19).

In the field of metabolomics, direct analysis in real time coupled to high resolution mass spectrometry (DART-HRMS) is considered an innovative, ambient mass spectrometric approach, successfully applied in clinical screening, microbiology, food safety, and toxicology (20–30). DART-HRMS requires minimal sample preparation and it has already demonstrated its accuracy, intra-sample repeatability and rapidity, with the aim of reducing the burden of chemical laboratories. In addition, DART-HRMS allows screening the metabolic profile without any prior knowledge of the identity of the metabolic features or their physicochemical characteristics (31). In parallel with genomic and transcriptomic analysis (11, 14–16, 32), this non-targeted approach produces a large amount of information within a very short time by the application of different extraction procedures and instrumental modalities. Statistical strategies called data fusion can then be applied to adequately integrate the data into a unique global dataset that can be submitted to multivariate analysis (33, 34).

In the present study, sera collected from MAP infected, infectious and negative animals were submitted to polar and non-polar extraction, then analyzed by DART-HRMS in positive and negative ion modes. Latent variables extracted from partial least squares discriminant analysis (PLS-DA) of single datasets were merged and submitted to a fused-PLS-DA with the aim of teasing out the characteristic molecular markers of the MAP infected, infectious and negative status.

## Materials and Methods

### Animal Selection and Time Course of the Study

Holstein cattle were selected from four dairy farms with known paratuberculosis initial seroprevalences of >10% and were divided into age-cohorts by reproduction cycle: heifers, primiparous, and pluriparous cows. A total of 356 animals were monitored up to 4 lactations, with blood and fecal sample collections at 30 ± 15 days before the expected calving date to minimize individual metabolic variations except for young heifers that were recruited at 13–15 months of age. During the pre-calving period, cows do not produce milk and the metabolic-hormonal changes that prepare the animal for calving are not established yet. MAP affected animals and cattle in bad health condition were excluded from the study. Blood sample collection was performed under authorization n. 506/2015 of the Italian Ministry of Health for the use of animals for experimental purposes.

### Sample Collection and Testing for JD

Blood samples were collected from the jugular vein in an anticoagulant free vacutainer tube, left to coagulate at room temperature for 2–4 h and centrifuged at 2,500 rpm for 5 min. Aliquots from the sera obtained were used for detecting serum antibodies against MAP using a commercial enzyme-linked immunosorbent assay (ELISA) (IDEXX Paratuberculosis Screening Ab, IDEXX Laboratories, Inc.) and applying the manufacturer's instructions for analysis. Two other aliquots of each serum were stored at −80°C for metabolomic analyses and for possible future analyses. Suspect and positive sera were submitted to an ELISA biphasic confirmation test (IDEXX Paratuberculosis Verification Ab, IDEXX Laboratories, Inc.).

Individual fecal samples were collected from the rectal ampulla and analyzed applying microbiological and molecular diagnostic methods for MAP identification. One fecal aliquot from each animal was stored at −80°C for possible future analyses.

All samples were processed for testing by direct real-time PCR (qPCR) and culture on modified Middlebrook 7H9 liquid media (7H9+). In brief, 2 g of feces were resuspended in 10 ml of water; mixtures were rocked on a horizontal shaker for 30 min and left to sediment for an additional 30 min. At the end of this step, an aliquot of 300 μl of supernatant was collected for PCR testing and 5 ml were processed for culture following a modified double-decontamination and centrifugation method (35). In brief, the supernatant was resuspended in 10 ml of 0.75% hexadecylpyridinium, incubated overnight at 37°C, centrifuged, resuspended in PANTA antimicrobial mixture and reincubated overnight at 37°C. The next day, 200 μl were inoculated into two 7H9+ tubes and incubated at 37°C for 6 weeks. At the end of the period, the 7H9+ cultures were examined by Ziehl-Nielsen staining and the broth cultures were confirmed by real-time PCR.

The same PCR protocol was used for both confirmatory test and direct analysis. In brief, 300 μl of supernatant or culture medium was subjected to a bead-beating step in order to enhance MAP DNA recovery as previously described (36). DNA extraction was performed manually with the High Pure PCR preparation kit (Roche Diagnostic, Mannheim, Germany) or by the automated MagMAX™ 96 Viral Isolation Kit (Ambion, Austin, USA) using the Microlab Starlet automated extraction platform (Hamilton Robotics, Bonaduz, Switzerland) or the KingFisher Flex instrument (ThermoFisher Scientific Inc., Worcester, MA, USA). In all cases, DNA extraction was performed according to the manufacturer's instructions. Real-time amplification was performed in a 7300 Real-Time PCR System (Applied Biosystems, Nieuwerkerk a/d IJssel, The Netherlands) or a CFX96 Touch Deep Well real-time PCR system (Bio-Rad Laboratories, Segrate, Italy). The amplification mixture contained 900 nM of each primer, 200 nM of probe, and 1X 1× Taq GOLD PCR Master Mix (Applied Biosystems) in a volume of 25 μl. The primers (Map668F-5′-GGCTGATCGGACCCG-3′, Map791R-5′-TGGTAGCCAGTAAGCAGGATCA-3′) and probe (Map718 5′-FAM-ATACTTTCGGCGCTGGAACGCGC-TAMRA) were designed on a MAP-specific portion of IS900. The real-time PCR program was 2 min at 50°C followed by 10 min at 95°C, 40 cycles at 95°C for 15 s and 60°C for 1 min. A cut-off value of 38 cycles was set according to the laboratory's validation procedure (35).

### JD Health Status Assignment and Sample Selection for DART-HRMS Analysis

Out of 356 animals, a total of 854 samples were collected during the study period resulting in a mean value of 2.40 samplings per cattle (range 1–5). Regarding JD testing, the frequency of positive animals along the study period resulted to be 6.23% by serology and 11.05% by fecal PCR/culture. At the end of the prospective study, the infectious status was assigned to a serum sample based on a seroconversion for MAP by ELISA accompanied by a positive result to fecal PCR or culture. The infected status was retrospectively assigned to the previous sampling of the animals classified as infectious, in which all JD tests (ELISA, PCR and culture) produced negative results.

The negative status was eventually assigned to exposed cohort animals selected from the same infected herds with the presence of at least one subsequent JD negative result after the selected sampling. These control animals were matched to cases according to sampling date and age category (heifers, primiparous cows, and pluriparous cows) in order to minimize the variability due to dietary and management conditions. The average number of samplings for these animals was 2.84 (range 2–4).

From the collection of sera stored at −80°C, 17 sera of infectious animals, 10 sera of infected animals and 20 sera of negative animals were selected and then analyzed by “DART-HRMS.” The age of the selected animals averaged 51.4 months (range 13–119 months).

### DART-HRMS Analysis

#### Sample Extraction

Frozen aliquots of sera were thawed and submitted to two different extraction procedures. In the first protocol, 200 μL of serum were diluted in 800 μL of water and methanol (H_2_O:MeOH; 20:80 v/v) solution (MilliQ water and methanol HPLC-grade with 99.9% purity, from VWR International, Radnor, USA), vortexed for 30 s, sonicated for 15 min, and centrifuged for 5 min at 12,000 rpm. In the second extraction procedure, 200 μL of serum were diluted in 800 μL of pure ethyl acetate (EtAc) (99.9% purity, Carlo Erba Reagents, Cornaredo, Italy), vortexed for 30 s, sonicated for 15 min, and centrifuged for 5 min at 12,000 rpm.

#### DART-HRMS

The instrumental analyses were carried out using a DART SVP 100 ion source (IonSense, Saugus, USA) coupled with an Exactive Orbitrap (Thermo Fisher Scientific, Waltham, USA). The DART source was coupled with a Dip-it^(R)^ sampler (IonSense, Saugus, MA, USA). A Vapur interface (IonSense, Saugus, USA) facilitated the passage of the ions from the DART source to the mass spectrometer. The distance between the DART gun and the ceramic transfer tube of the Vapur interface was 12 mm. The optimized DART settings were as follows: grid voltage 250 V, temperature 300°C, sample speed 0.3 mm/s, and a single time analysis of 0.66 min. The system parameter settings for the mass spectrometer were as follows: 55 S-lens RF level, capillary temperature 300°C, and maximum injection time 10 ms. The resolution was set to 70.000 FWHM and the mass range was 50–1,000 Da in both positive and negative ion modes. In positive ion mode, a vial with an aqueous solution of 25% ammonia was positioned below the DART gun exit, working as a dopant to facilitate and stabilize the formation of [M+NH_4_]^+^ ions.

All DART-MS analyses were run with an automated gain control target setting of 3 × 10^6^. Homemade Dip-it tips (IonSense, Saugus, USA) were inserted into a holder of the autosampler, and then 5 μL of each extract were spotted onto each Dip-it tip. Subsequently, the Dip-it tips automatically moved at a constant speed of 0.3 mm/s throughout the DART gun exit and ceramic tube of the Vapur interface. The duration of desorption from the surface of each tip was about 20 s.

The samples were analyzed in triplicate and XCalibur QualBrowser software (Thermo Fisher Scientific, Waltham, USA) was used to visualize the entire spectra in raw format. These were converted to mzML files using Proteowizard and then opened with mMass software (http://www.mmass.org/), which allowed interpretation of the mass spectrometry data and assignment of ions using the online METLIN (https://metlin.scripps.edu) and HUMAN METABOLOME DATABASE (www.hmdb.ca) libraries. Prior to statistical analysis, the spectra of the four datasets (two extraction solvents *per* two ion modes) were converted into.csv files with Rstudio 3.6.1 software (RStudio Team, 2016; RStudio Integrated Development for R; RStudio, Inc., Boston, USA).

### Data Processing and Statistical Analysis

The DART-HRMS spectral data, acquired in triplicate and not averaged, were statistically analyzed using MetaboAnalyst 4.0 web platform (www.metaboanalyst.ca) and Rstudio 3.6.1 software (37). The three replicates were used independently as done previously in several chemometric studies (38, 39). The signals of the four datasets were loaded onto MetaboAnalyst 4.0 web platform and aligned with a tolerance of 0.008 Da. We removed the ion signals with more than 75% of missing values (no detected ion intensity) over all the samples. The ions with <75% of missing values had them replaced with 1/5 of the recorded lowest intensity (40). Isotopes were removed. Normalization by sum was applied to the signals, whereby each feature was normalized by Pareto scaling. First, supervised partial least squared discriminant analysis (PLS-DA) was performed on the four separate datasets (2 extraction solvents × 2 instrumental ion modes) to evaluate the possible improved discrimination achieved by mid-level data fusion (Supplementary Figures 1–4).

A low level data fusion was also tested and described in the Supplementary Material (Supplementary Figure 5).

#### Mid-Level Data Fusion

Using Rstudio 3.6.1 software, the first five PLS-DA score components of each dataset were fused (concatenated) and submitted to a new PLS-DA with the aim at discriminating the three groups of samples (30). Evaluating the *R*^2^, *Q*^2^, and accuracy of each separate PLS-DA, (–)DART-HRMS spectra of polar extraction were excluded because they provided poor information. Mid-level data fusion of the three most informative datasets was performed and the merged data submitted to fused-PLS-DA clustering (41). Ten-fold cross-validation was performed on the entire dataset to evaluate the performance of the fused-PLS-DA and to select the best number of components within the loading tables of each separate PLS-DA. As recommended by Borràs et al. (41), to extract the PLS-DA features, the best number of components (5) was chosen when the classification error obtained by cross-validation was minimized.

**Figure 1:**
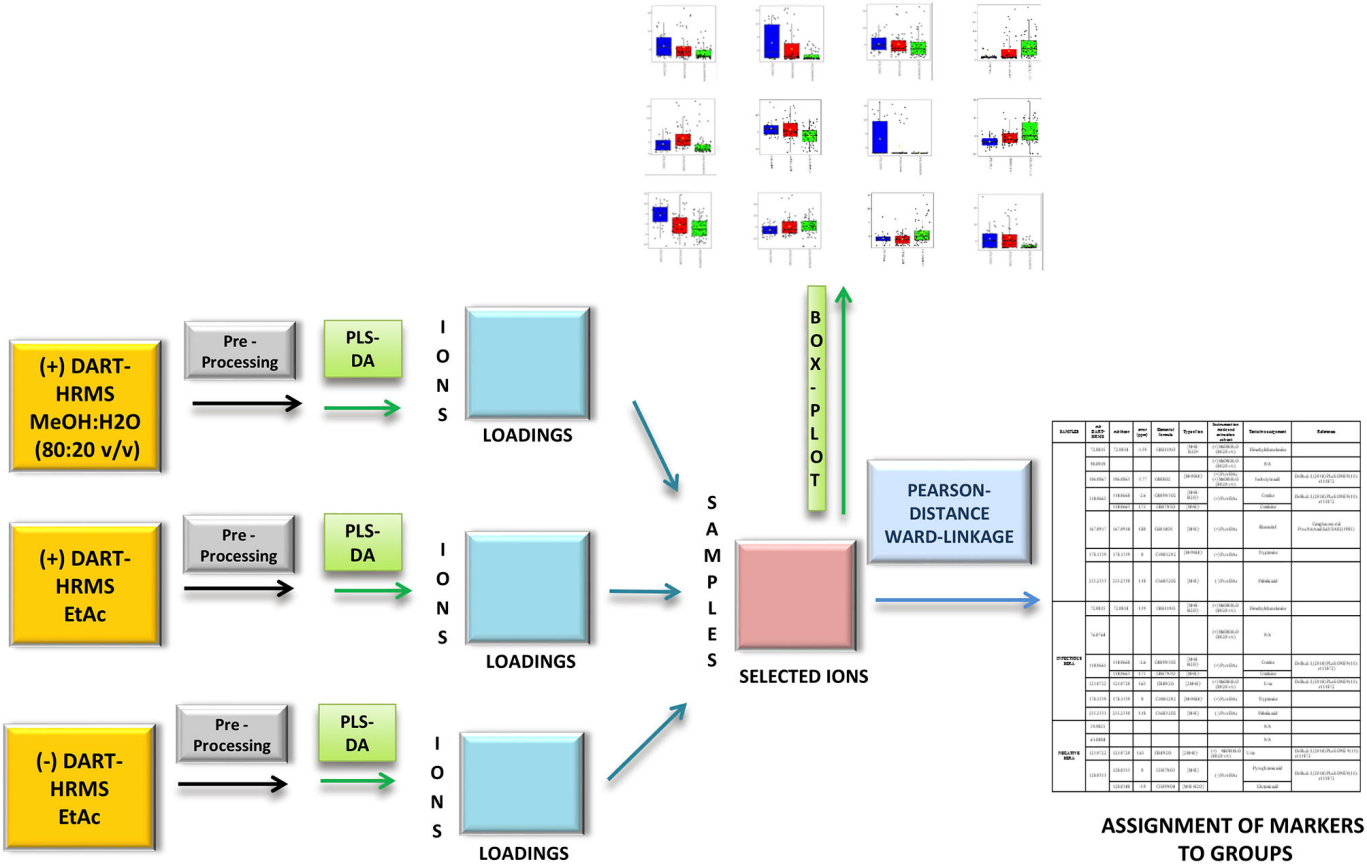
Flow chart of the mid-level data fusion and consequent statistical analysis of the three most informative (+/–) direct analysis in real time high resolution mass spectrometry (DART-HRMS) datasets with the aim selecting the most discriminative variables. After DART-HRMS data pre-processing (gray boxes), three exploratory partial least squares discriminant analysis were carried out (green boxes) and their most discriminative loadings (threshold value higher than 0.3) were selected and merged (red box). Box plots that denote differences in normalized ion abundance of biomarker ions were extrapolated from pre-processed data (orange box). A hierarchical cluster analysis (HCA) by the Pearson's distance criterion and Ward linkage allowed markers to be correlated to groups.

Finally, only the ions whose loadings had an absolute value higher than 0.3 were retained from the first five components of the three separate PLS-DA (Figure 1, blue boxes). A table with the samples in the rows and the selected significant ions in the columns was built (Figure 1, pink box). The obtained table was used to calculate Pearson's distance and Ward linkage to determine the correlation of the selected ions among the three JD health status groups.

## Results

In this study, a dual mode DART-HRMS analysis was performed on sera extracted using polar and non-polar solvents with the aim of finding the changes in metabolites' level in MAP-infected and infectious cattle as compared to those negative. Four DART-HRMS spectral datasets were thus acquired. Once pre-processed, each dataset was submitted to PLS-DA.

To overcome the difficulty of handling high dimensional datasets, a data reduction was conducted to fade out the most significant variables capable of discriminating the three MAP status groups. To this aim, a mid-level data fusion strategy was attempted by operating at the level of features and thus capturing only the relevant differences in the four datasets (33, 41, 42). In this case, good separation was obtained within the PC1 vs. PC2 score space (Supplementary Figure 6), explaining 32.9 and 10.6% of the total variance of the model. Although we obtained a good separation, we removed the less informative dataset.

**Figure 2:**
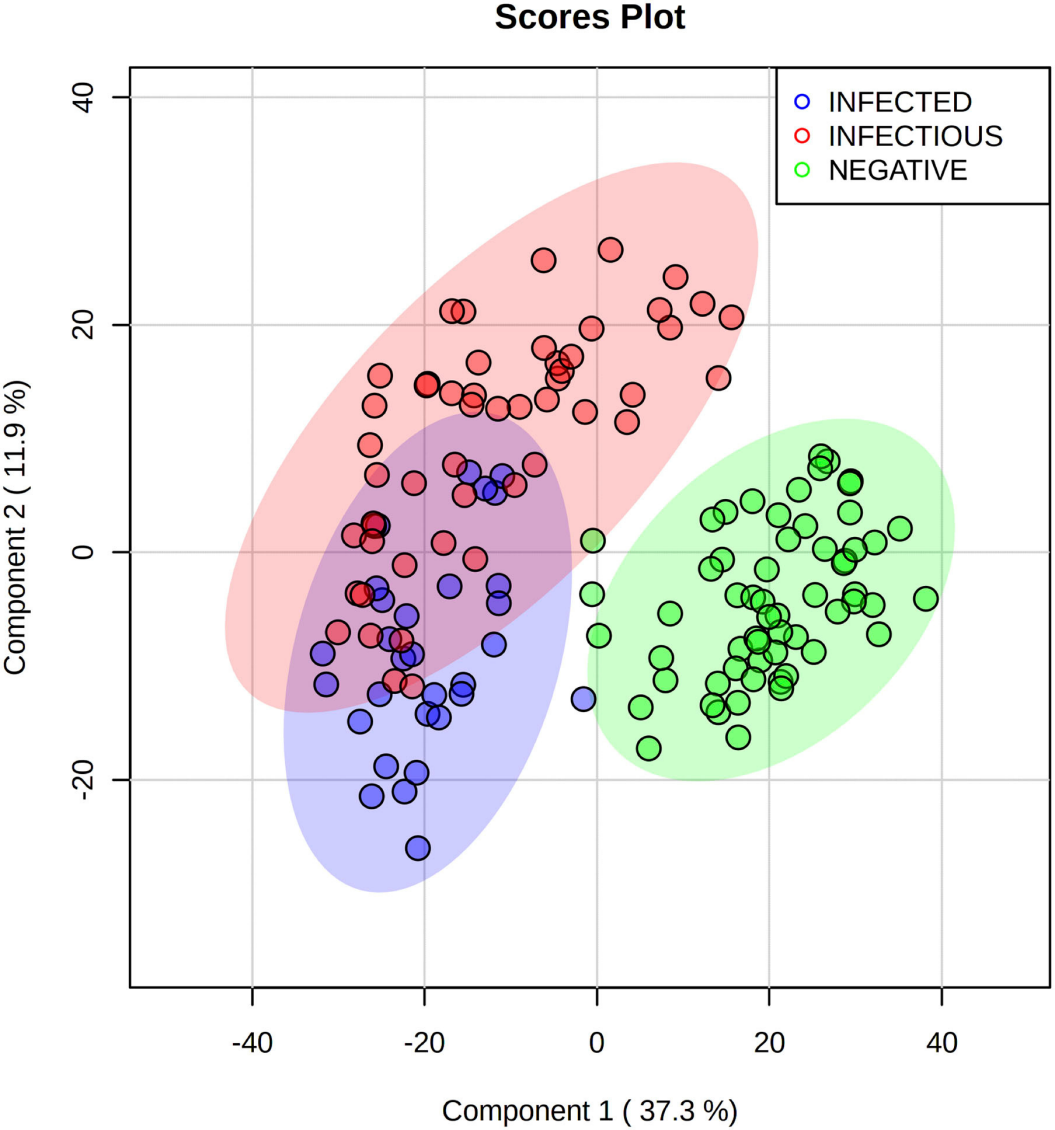
Partial least squares discriminant analysis score plot of the three most informative Direct analysis in real-time high-resolution mass spectrometry datasets merged by mid-level data fusion. MAP infected (blue) and infectious (red) cattle are successfully discriminated from negative (green) cattle. A slight overlap can be seen between infected and infectious groups. Three repetitions for each sample were used.

To further improve the discrimination, we evaluated the *R*^2^, *Q*^2^, and accuracy of each separate PLS-DA (data not shown) and realized that (–)DART-HRMS of polar extractions provided no informative data for the discrimination of the groups. Hence, we merged the scores of the three most informative datasets and performed a PLS-DA. The score plot showed an improvement in clustering (Figure 2), with most samples from the MAP negative group correctly assigned to their 0.95-ellipsis confidence interval, and some samples that overlapped between the infected and infectious groups. In this case, PC1 vs. PC2 explained 37.3 and 11.9% of the total variance of the model.

The 12 most informative *m/z* values were extrapolated by selecting the loadings with absolute value higher than 0.3. They were then fused in a single dataset and submitted to Pearson's distance and Ward linkage calculation. In this way, correlation between ions and MAP status groups was obtained. Table 1 reports the *m/z* values that codified each MAP status group in terms of the most abundant molecular features. Representative DART-HRMS spectra showing the most informative *m/z* values are reported in the Supplementary Material (Supplementary Figures 8–10).

**Table 1:** List of discriminant (+/–) direct analysis in real time high resolution mass spectrometry metabolites observed in sera from groups of MAP infected, infectious and negative cattle.

**Samples**	***m/z* DART-HRMS**	***m/z* theor**	**error (ppm)**	**Elemental formula**	**Type of ion**	**Instrument ion mode and extraction solvent**	**Tentative assignment**
**INFECTED SERA**	72.0815	72.0814	−1.39	C_4_H_11_N_O_	[M+H–H_2_O]^+^	(+) Pure EtAc	Dimethylethanolamine
	90.0919					(+) MeOH:H_2_O (80:20 v/v)	N/A
	106.0867	106.0863	−3.77	C_4_H_8_O_2_	[M+NH_4_]^+^	(+) Pure EtAc	Isobutyric acid^+^
						(+) MeOH:H_2_O (80:20 v/v)	
	114.0665	114.0668	−2.6	C_4_H_9_N_3_O_2_	[M+H–H_2_O]^+^	(+) MeOH:H_2_O (80:20 v/v)	Creatine^+^
		114.0663	1.75	C_4_H_7_N_3_O	[M+H]^+^		Creatinine^+^
	167.0917	167.0914	1.80	C_6_H_14_O_5_	[M–H]^+^	(+) Pure EtAc	Rhamnitol
	178.1339	178.1339	0	C_10_H_12_N_2_	[M+NH_4_]^+^	(+) MeOH:H_2_O (80:20 v/v)	Tryptamine
	255.2333	255.2330	1.18	C_16_H_32_O_2_	[M–H]^−^	(-) Pure EtAc	Palmitic acid (16:0)
**INFECTIOUS SERA**	72.0815	72.0814	1.39	C_4_H_11_NO	[M+H–H_2_O]^+^	(+) MeOH:H_2_O (80:20 v/v)	Dimethylethanolamine
	76.0764					(+) MeOH:H_2_O (80:20 v/v)	N/A
	114.0665	114.0668	−2.6	C_4_H_9_N_3_O_2_	[M+H–H_2_O]^+^	(+) Pure EtAc	Creatine^+^
		114.0663	1.75	C_4_H_7_N_3_O	[M+H]^+^		Creatinine^+^
	121.0722	121.0720	1.65	CH_4_N_2_O	[2M+H]^+^	(+) MeOH:H_2_O (80:20 v/v)	Urea dimer^+^
	178.1339	178.1339	0	C_10_H_12_N_2_	[M+NH_4_]^+^	(+) Pure EtAc	Tryptamine
	255.2333	255.2330	1.18	C_16_H_32_O_2_	[M–H]–	(–) Pure EtAc	Palmitic acid (16:0)
**NEGATIVE SERA**	59.9855					(–) Pure EtAc	N/A
	61.0404					(+) MeOH:H_2_O (80:20 v/v)	N/A
	121.0722	121.0720	1.65	CH_4_N_2_O	[2M+H]^+^	(+) MeOH:H_2_O (80:20 v/v)	Urea dimer^+^
	128.0353	128.0353	0	C_5_H_7_NO_3_	[M–H]^−^	(–) Pure EtAc	Pyroglutamic acid^+^
		128.0348	−3.9	C_5_H_9_NO_4_	[M–H–H_2_O]^−^		Glutamic acid^+^

*The m/z values, theoretical mass, error (ppm), elemental formula, type of ion, ion mode and extraction procedure, tentative assignment, and literature references are reported*.

+ *(13)*.

**Figure 3:**
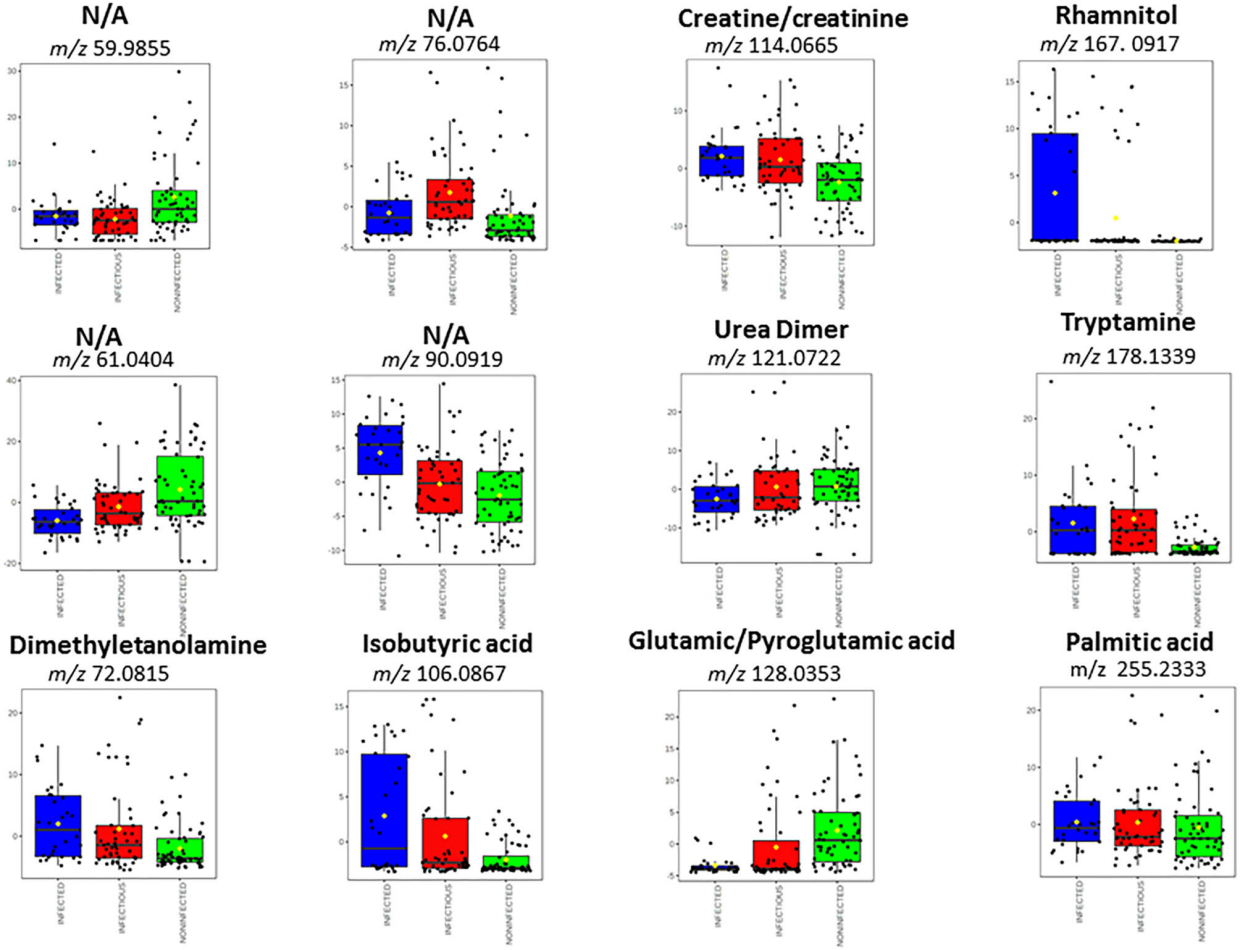
Box plots that denote differences in normalized ion abundances for markers that contributed to the statistical discrimination between MAP infected, infectious and negative animals. The box plot diagrams were retrieved from Metaboanalyst web portal and used without further modification. The bottom and top of the boxes represent the 25th and 75th percentiles, the line in the middle indicates the 50th percentile or the median. The black circles represent the entire data range, including the extreme value outliers not taken into consideration. Three repetitions for each sample were used.

Figure 3 shows box plots with the relative intensities of the observed biomarkers. Sera of infected cattle presented higher relative abundances (compared to the other MAP groups studied) of dimethylethanolamine (*m/z* 72.0815 in positive ion mode), isobutyric acid (*m/z* 106.0867 in positive ion mode), creatine a/o creatinine (*m/z* 114.0665 in positive ion mode), rhamnitol (*m/z* 167.0917 in positive ion mode), tryptamine (*m/z* 178.1339 in positive ion mode), palmitic acid (*m/z* 255.2333 in positive ion mode), and the unassigned ion, *m/z* 90.0919. The serum profiles of infected cattle were also characterized by lower relative intensity of urea (the dimer of *m/z* 121.0722 in positive ion mode). In addition, the molecular signature of sera from infectious cattle showed higher relative abundance of dimethylethanolamine (*m/z* 72.0815 in positive ion mode), isobutyric acid (*m/z* 106.0867 in positive ion mode), creatine a/o creatinine (*m/z* 114.0665 in positive ion mode), tryptamine (*m/z* 178.1339 in positive ion mode), and the unassigned ions, *m/z* 76.0764 and *m/z* 90.0919.

Compared to sera of infected and infectious animals, negative cattle showed higher levels of pyroglutamic acid a/o glutamic acid (observed in negative ion mode at *m/z* 128.0353), urea (*m/z* 121.0722), and the unassigned ions of *m/z* 61.0404 and 59.9855.

To further investigate the discriminatory capacity of the selected biomarkers, internal cross-validation was performed on the PLS-DA of the three most informative DART-HRMS datasets after fusion. The outcomes of the cross-validation, expressed as accuracy (0.91), *Q*^2^ = 0.57, and *R*^2^ = 0.48, are summarized in Supplementary Figure 7. The accuracy, with a maximum theoretical value of 1, is the capability of the model to correctly classify the samples. While the *R*^2^ parameter indicates how well the PLS-DA model explains the current dataset, *Q*^2^ provides a qualitative measure of consistency between the predicted and actual data (43). The parameters *Q*^2^ and *R*^2^ each have a theoretical maximum of 1 and acceptable values of ≥0.4 for a biological model (44). Note that in metabolomics, the values of these parameters strongly depend on the individuals that constitute the validation subsets. Since cross-validation requires systematic deletion of large portions of the dataset during training, non-trustable values of *Q*^2^ can be produced (44, 45). Since no large difference between *R*^2^ and *Q*^2^ values was observed in the present study, overfitting can be excluded (46).

## Discussion

In metabolomics, the selection of the most appropriate statistical analysis method allows extrapolation of significant features that can characterize each group in terms of its metabolic content. In a non-targeted approach, statistical analysis is often employed in two modes: (i) “model building,” for the purpose of unravel important molecular markers that describe each group of samples, and; (ii) “classification,” for the purpose of sample assignment through matching its spectrum to a previously built model.

In the present study, characterized by small sized and slightly unbalanced groups, we focused on model building aiming to tease out and identifying informative *m/z* values capable of describing each group (i.e., the infected, infectious and negative animals in infected herds.

We applied multimodality DART-HRMS that allows more comprehensive information to be obtained, although with the counterpart of its over-dispersion in the case of big dimensional data. To overcome this issue, we exploited a mid-level data fusion strategy, which demonstrated good discriminatory power. We confirmed that this technique can catch the existent variability among groups and that compression of the high dimensional data was imperative to fade out the existent correlations between markers (33, 34). DART-HRMS coupled to statistical analysis demonstrated its capability to discriminate the negative group from infected and infectious cattle. On the other hand, this approach provided a tendency to discrimination between MAP infected and infectious animals due to a partial superposition of their metabolic profiles (Figure 2 and Supplementary Figures 8–10).

MAP infected and infectious cattle had higher serum levels of creatine/creatinine, palmitic acid, dimethylethanolamine and tryptamine than did negative cattle.

The high creatine/creatinine abundances, already reported in experimental MAP infection (13), could be associated with muscle wasting due to the protein catabolism determined by the disease progression the led to a reduced energetic intake (47). In addition, we observed a higher abundance of palmitic acid in both MAP infected and infectious cattle than in negative animals. Alterations in the synthesis and absorption of phospholipids and sphingolipids presumably contribute to the circulation of high levels of the free fatty acid. In this context, Wood et al. found significant decrease in circulating levels of phospholipids and sphingolipids probably due to the dysfunction of the gastrointestinal (GI) epithelium and the liver (17). Furthermore, Thirunavukkarasu *et al* demonstrated the altered expression of genes associated with cholesterol and lipid metabolism (48). In parallel, in patients with active *Mycobacterium tuberculosis* infection, phosphatidylglycerol (PG 16:0_18:1), the structural components of which are oleic acid and palmitic acid, was one of the molecules significantly elevated in the plasma (49). In the present study, the hydrolysis of phospholipids could have occurred during the extraction procedure or DART ionization.

In the same vein, high relative abundances of dimethylethanolamine were found in sera of both infected and infectious cattle. Dimethylethanolamine is a structural analogue of choline that is involved in the metabolism of phospholipids, absorption of which can be altered by gastrointestinal microbiome alteration (50). High levels of tryptamine (involved in tryptophan metabolism), observed in both infected and infectious animals, can be explained by important changes in protein turnover or deficiencies in MAP infected cattle (13). Interestingly, higher levels of isobutyric acid were measured in infected animals than in negative animals (Table 1 and Figure 3). Higher relative intensities of isobutyric acid in infected than in negative sera could be related to inflammation of the gut due to MAP infection.

The lower urea level in MAP-infected cattle has already been reported by De Buck et al. (13), who interpreted this phenomenon as being due to increased muscle turnover, likely altering amino acid metabolism and reducing energy intake.

Interestingly, while De Buck et al. (13) observed increased glutamic acid in both MAP infected and control groups, due to the influence of developmental and diet changes of the life of the calves, we observed higher glutamic/pyroglutamic acid levels only in sera of negative animals. Glutamic acid is involved in energy metabolism, immunity and GI function. Its scarcity in infected and infectious animals could be explained by a low immune response when cattle are in the advanced JD stages and by the damage of GI system. Since glutamic acid is also produced by muscles, low serum levels could also be associated with muscle wasting.

Finally, pyroglutamic acid and glutamic acid are involved in glutathione (GSH) metabolism. Pyroglutamic acid is a downstream metabolite of GSH metabolism, and glutamic acid is involved in the synthesis of GSH. GSH is an important antioxidant that protects cells from oxidative stress. Both were lower in the sera of infected and infectious cattle than in that of negative cattle, suggesting that MAP alters GSH metabolism.

In conclusion potential caveats of this prospective field study on natural MAP infection in dairy cattle must be taken into account. Considering the long MAP incubation period, we do not exclude that negative animals could have changed their status after the latest sampling carried out at least 1 year after the selected time-point.

Note that we made any possible effort to reduce metabolic variability due to season, feeding and management, by: (i) testing all animals (except young heifers) during the pre-calving period and (ii) matching negative animals to cases by sampling date and age category. Finally, individual differences could have affected the results because of the small sized groups.

## Conclusion

The main goal of mid-level data fusion is to optimize high dimensional information, exploit the synergies of information provided by different analytical strategies, and fade out the existent correlations between markers and groups. The construction of this model using DART-HRMS data revealed the characteristic serum metabolites of MAP infected, infectious, and negative cattle. The model, built by fusing the three most informative DART-HRMS datasets, produced a reasonably high degree of accuracy, suggesting the markers are capable of discriminating the health status of cattle with regard to MAP infection. In the future, the model will be populated with results from more animals in order to improve its robustness and performance. Proper validation of the model will be performed to evaluate its discriminative capacity and verify its potential for diagnostic purposes. Prospectively producing an earlier diagnosis, it will then be easier to manage infected cattle, prevent the diffusion of the pathogen and, thus, control JD on dairy farms.

## Data Availability Statement

The raw data supporting the conclusions of this article will be made available by the authors, without undue reservation.

## Ethics Statement

The animal study was reviewed and approved by authorization n. 506/2015 of the Italian Ministry of Health. Written informed consent was obtained from the owners for the participation of their animals in this study.

## Author Contributions

NP, RP, LP, and JD designed the research. BM, MB, PF, MD, and AS performed the experiments. LP contributed to sampling. AM performed the statistical analyses. AT, IP, and AM interpreted the data and wrote the manuscript. NP, RP, and JD interpreted the results and edited the manuscript. All contributed to the editing the manuscript.

## Conflict of Interest

The authors declare that the research was conducted in the absence of any commercial or financial relationships that could be construed as a potential conflict of interest.
