# Supplementary material for: Serum Metabolomic Profiles of Paratuberculosis Infected and Infectious Dairy Cattle by Ambient Mass Spectrometry
Source: Front Vet Sci. 2021 Jan 20;7:625067. doi: 10.3389/fvets.2020.625067 (PMC7854907; doi:10.3389/fvets.2020.625067)
Supplement: Supplementary file 1 [file Data_Sheet_1.docx]

Supplementary Material

*1.Exploratory statistical analysis on separate DART-HRMS datasets:*

Four DART-HRMS spectral datasets were thus acquired. Once pre-processed, each dataset was submitted to supervised partial least squared discriminant analysis (PLS-DA). A 10-fold cross validation was performed to evaluate the performance of each PLS-DA. In 10-fold cross-validation, the original samples were randomly split into two equal sized groups. The first group of subsamples was retained as the validation set for testing the model, and the remaining subsamples were used to train the model. The cross-validation process was eventually repeated 10 times. Unfortunately, no clear clustering was observed as shown in **Figures S1-S4**.

**
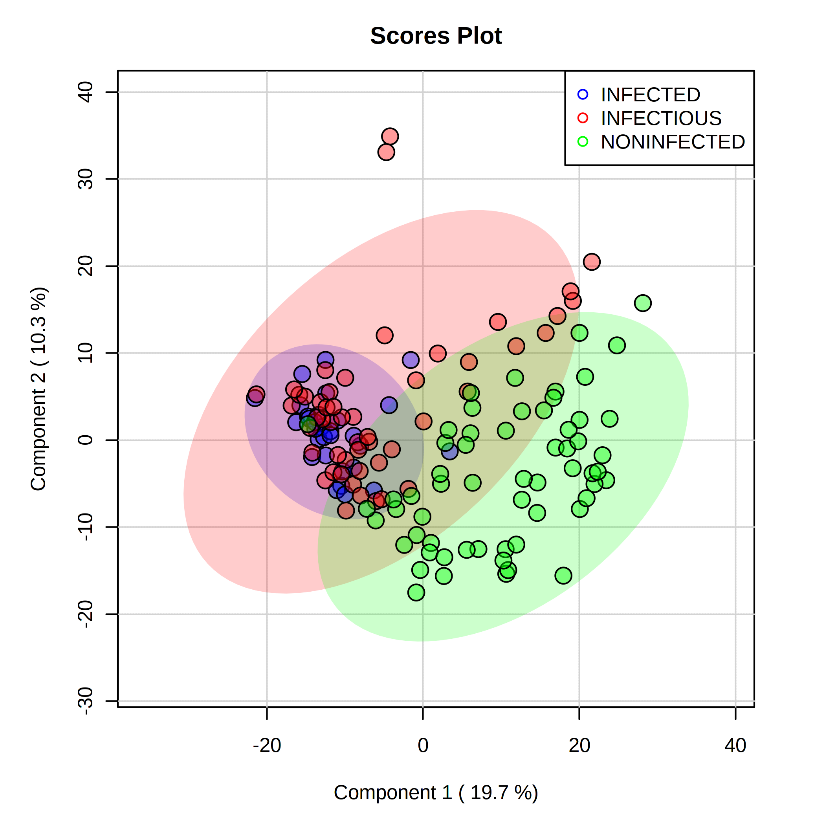
**

INFECTED

INFECTIOUS

NEGATIVE

**Supplementary Figure 1.** PLS-DA score plot of MAP-infected, MAP-infectious and negative sera using (-) DART-HRMS spectra of non- polar extracts. No discrimination was achieved

INFECTED

INFECTIOUS

NEGATIVE


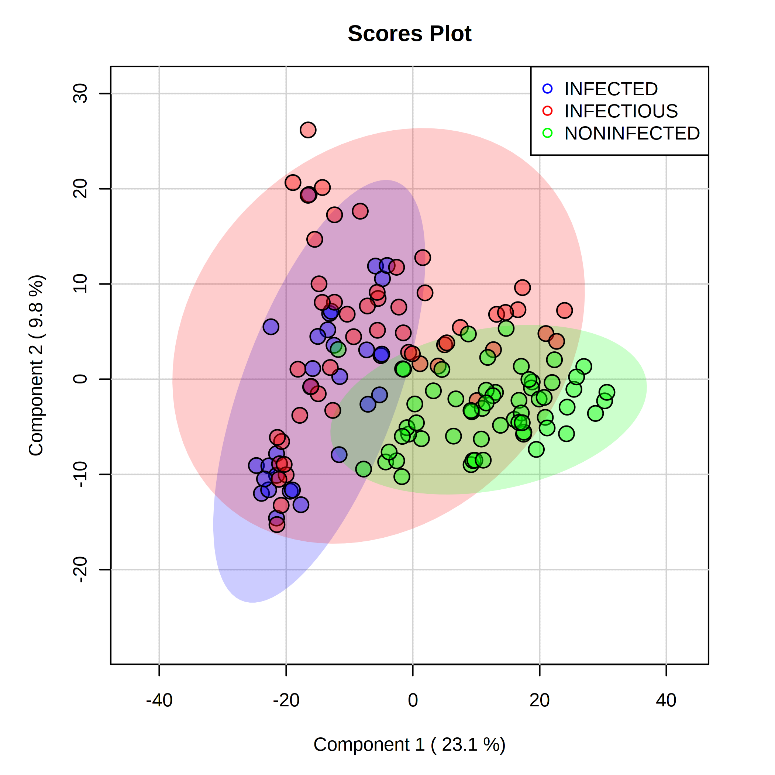


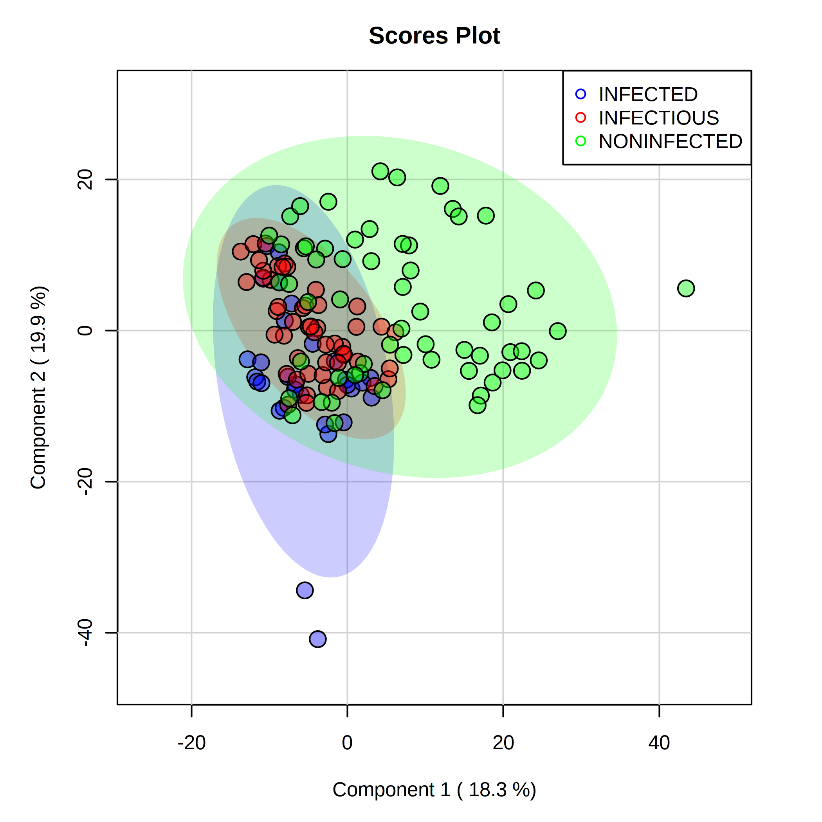
**Supplementary Figure 2.** PLS-DA score plot of MAP-infected, MAP-infectious and negative sera using (+) DART-HRMS spectra of non- polar extracts. No discrimination was achieved.

INFECTED

INFECTIOUS

NEGATIVE

**Supplementary Figure 3.** PLS-DA score plot of MAP-infected, MAP-infectious and negative sera using (-) DART-HRMS spectra of polar extracts. No discrimination was achieved.


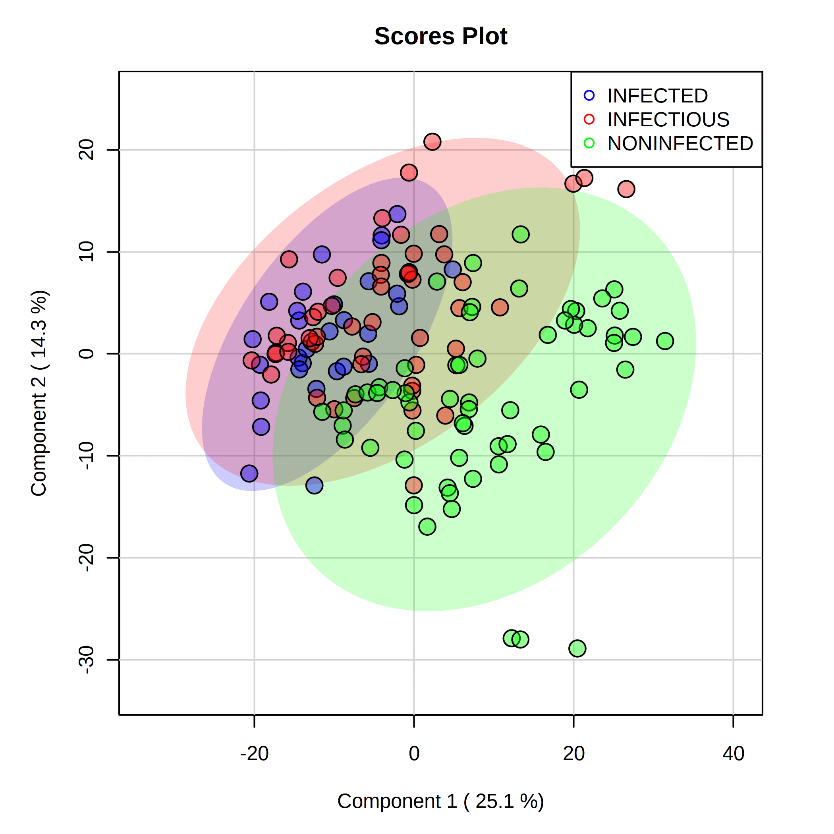


INFECTED

INFECTIOUS

NEGATIVE

**Supplementary Figure 4.** PLS-DA score plot of MAP-infected, MAP-infectious and negative sera using (+) DART-HRMS spectra of polar extracts. No discrimination was achieved.

*2. Low-Level Data fusion*

In order to get the most comprehensive biomarker lists capable of describing each group of sera, we decided to fuse the data from all four datasets using a low-level approach. PLS-DA of the fused datasets from positive and negative ion mode spectra of both non-polar and polar extracts also provided unsatisfactory discrimination (**Figure S5**).In low-level data fusion, pre-processed signals obtained from the four data matrices were merged and then processed as a unique fingerprint of the samples. PLS-DA of the merged datasets was performed to visualize relationships among groups and variables, considering the whole information content (obtained from two extraction solvents with two instrumental ion modes).


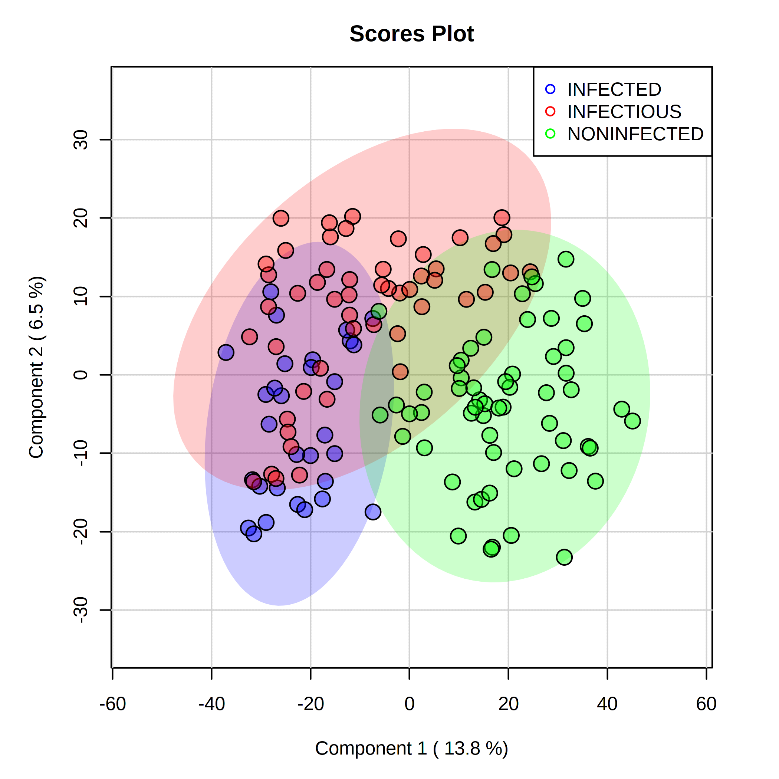


INFECTED

INFECTIOUS

NEGATIVE

**Supplementary Figure 5.** PLS-DA score plot after low-level data fusion of (+/-)DART-HRMS spectra of polar and non polar extracts of MAP-infected, MAP-infectious and negative sera. No discrimination was achieved.

INFECTED

INFECTIOUS

NEGATIVE


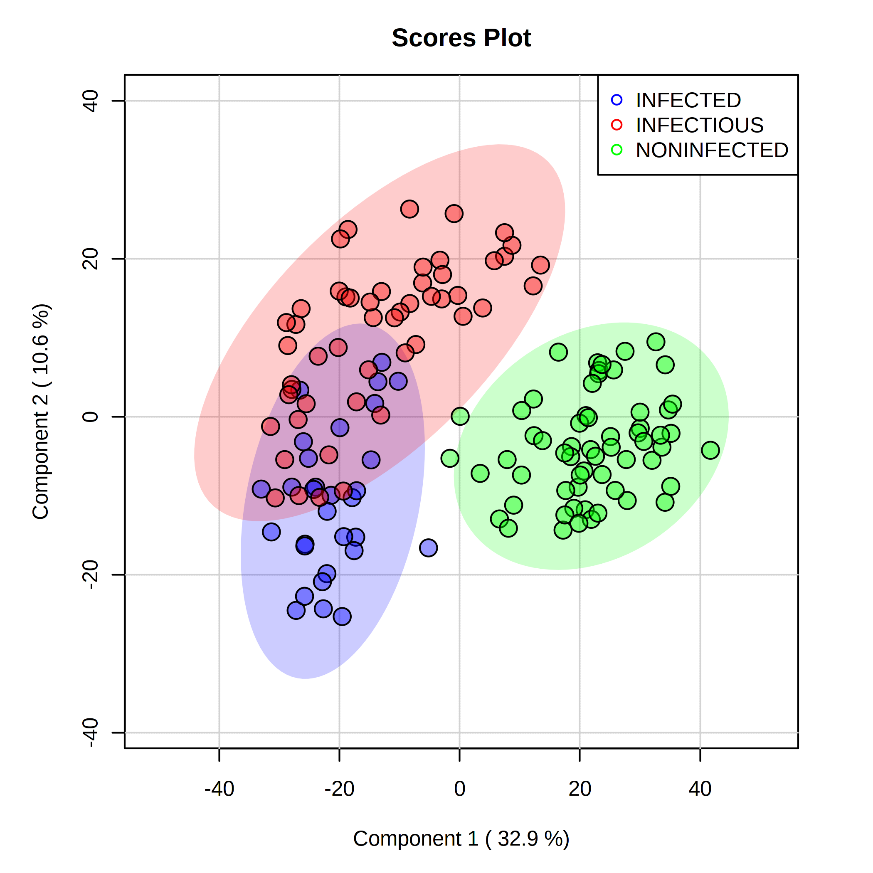


**Supplementary Figure 6.** PLS-DA score plot of MAP-infected, MAP-infectious and negative sera of merged (+/-)DART-HRMS of polar and non-polar extracts.

**
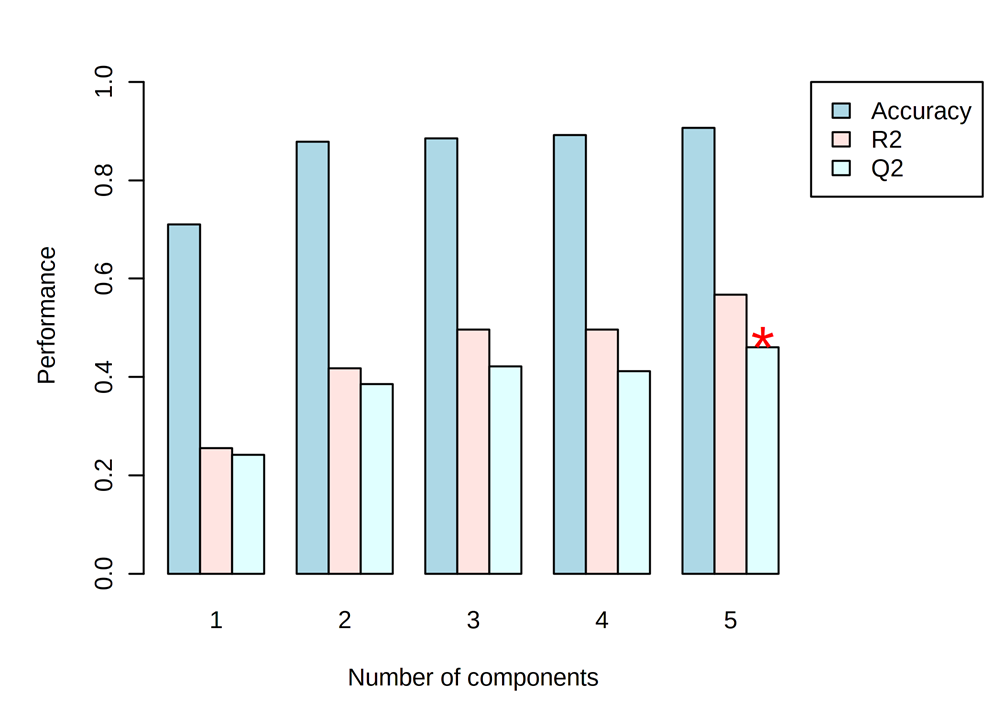
**

**Supplementary Figure 7.** Internal cross-validation was performed of the PLS-DA model built on the three most informative DART-HRMS datasets after mid-level data fusion. The outcomes of the cross-validation, expressed as accuracy =0.91, Q^2^=0.57, and R^2^=0.48

**Supplementary Figure 8.** Representative DART-HRMS spectra of MAP-infected sera. A) (+)DART-HRMS of polar extract of MAP-infected sera. B) (+)DART-HRMS of non-polar extract of MAP-infected sera. C) (-)DART-HRMS of non-polar extract of MAP-infected sera.

**Supplementary Figure 9.** Representative DART-HRMS spectra of MAP-infectious sera. A) (+)DART-HRMS of polar extract of MAP-infectious sera. B) (+)DART-HRMS of non-polar extract of MAP-infectious sera. C) (-)DART-HRMS of non-polar extract of MAP-infectious sera.

**Supplementary Figure 10.** Representative DART-HRMS spectra of negative sera. A) (+)DART-HRMS of polar extract of negative sera. B) (+)DART-HRMS of non-polar extract of negative sera. C) (-)DART-HRMS of non-polar extract of negative sera.
